# Ten-Year Trend in Emergency Department Visits for Sexually Transmitted Infections among Adolescents: A Retrospective Cross-Sectional Study in Italy

**DOI:** 10.3390/ijerph192114207

**Published:** 2022-10-31

**Authors:** Elena Viottini, Beatrice Albanesi, Elena Casabona, Roberta Onorati, Sara Campagna, Alberto Borraccino

**Affiliations:** 1 Department of Public Health and Paediatrics, University of Torino, 10126 Torino, Italy; 2 Epidemiology Unit, Local Health Unit TO3, 10095 Grugliasco, Italy

**Keywords:** sexually transmitted infections, adolescents, retrospective cross-sectional study, epidemiological trend

## Abstract

Sexually transmitted infections (STIs) are frequently underdiagnosed, representing a serious public health concern, especially during adolescence and in more vulnerable communities. Aim: to describe the last ten years of emergency department (ED) visits for STIs among adolescents. Methods: a retrospective cross-sectional observation was carried out in the Piedmont region in Italy. Data were retrieved through the Italian National Information System database. ED visits related to specific ICD-9-CM codes carried out on 11 to 19-year-old youths between 2011 and 2020 were investigated. Age-specific, crude, and standardized rates and admission ratios, with 95% confidence intervals (CIs), were calculated to estimate the STI trend. Results: from a total of 1,219,075 ED visits, 339 were related to STIs, representing an increasing ratio of 28 per 100,000 visits, primarily in females. Most infections occurred in girls (83.5%) and among 17 to 19-year-olds (71.5%). A drop in both ED visits and STI cases was observed in 2020. Genital Herpes and Genital Warts were more frequent in girls while Gonorrhea was more frequent in boys. Conclusions: the increasing trend of ED visits for STIs, particularly in girls, represents an emerging relevant public health issue that needs to be urgently tackled.

## 1. Introduction

According to the World Health Organization (WHO) more than 1 million sexually transmitted infections (STIs) occur every day worldwide among people from 15–49 years of age, and this number is growing steadily. STIs can be caused by more than 30 different bacteria, viruses, and parasites that are mainly spread by vertical transmission or horizontally through direct communication, mostly through sexual contacts [1].

In 2020, a global incidence of 374 million new cases can be mainly ascribed to four treatable pathogens: *Trichomonas vaginalis* (42%), *Chlamydia trachomatis* (34%), *Neisseria gonorrhoeae* (22%), and *Treponema pallidum* (2%). WHO data further indicates that almost 500 million adults are currently living with the *Herpes Simplex virus* (HSV) infection that causes Genital Herpes. Moreover, about 300 million women have the *Human Papilloma virus* (HPV) infection, the primary cause of cervical cancer and anal cancer among men who have sex with men [2]. In addition, there are emerging outbreaks of new infections that can be acquired by sexual contact, such as *Monkeypox*, *Shigella Sonnei*, *Neisseria Meningitidis*, *Ebola* and *Zika*, as well as re-emergence of neglected STIs such as *Lymphogranuloma Venereum* [1].

The Centers for Disease Control and Prevention (CDC) in the USA and the European Centre for Disease Control and Prevention (ECDC), for the European Countries, reported a gradual growth in Gonorrhea infection rates (overall 22% increase), Syphilis (15% increase) and Chlamydia (5.3% increase) between 2009 and 2018 [3,4,5,6].

Since most STIs are symptomless, they suffer a delay in diagnosis and remain frequently undertreated, giving rise to known complications, as well as increasing the risk of transmission [7]. Chlamydia represents the most common sexually transmitted infection in Europe [4], and together with other pathogens such as Gonorrhea, *Mycoplasma Genitalium* and *Trichomonas vaginalis* are widely studied as they are associated with various urogenital diseases such as urethritis, cervicitis and endometritis [8,9,10,11,12].

Unnoticed infections in the lower reproductive tract of women can lead to serious and long-term consequences if they ascend to the fallopian tubes and result in a Pelvic Inflammatory Disease (PID). A 30% increased risk of PID has been reported in women who tested positive for Chlamydia compared to those who tested negative [13].

As for the Italian regions, ECDC data reported that between 2014 and 2018 Gonorrhea and Syphilis had increased respectively from a crude notification rate of 1 to 1.5 cases and 1.9 to 2.5 cases every 100,000 inhabitants [3,6] and confirmed Chlamydia cases had also increased from crude number of 940 to 1206 cases [4]. Compared to the incidence reported for European Union, Italy ranked just below the esteemed average (4.7 new cases per 100,000). Since the last ten years, the incidence showed a slight increase in the 15–24 age group [14].

Vulnerable populations, such as adolescents, must be investigated to improve the prevention, treatment, and surveillance of STIs, and to guarantee tailored interventions [15]. The WHO and ECDC have drafted recommendations and developed strategies to strengthen the available monitoring systems [16,17].

In Italy, Gonorrhea, Syphilis, and *Pediculosis pubis* are the only mandatory notified infections, but this is not always complied with, leading to an underestimation of the number of occurring cases. Therefore, two sentinel surveillance systems were activated to cover this lack of data. Data are provided by outpatient centers which are specialized in the diagnosis and treatment of STIs, and by clinical microbiology centers, which report new cases of Chlamydia, Trichomonas, and Gonorrhea [14]. This network does not have national coverage and excludes data from other healthcare facilities such as emergency departments (EDs).

STIs are differently managed by general practitioners, family planning centers, outpatient or in inpatients specialized centers, depending on the patient’s choice or medical advice [18,19,20]. Seeking treatment for STIs is not a simple decision and is often combined with multiple factors partly personal and partly organizational.

In particular, specialized centers offer sex education, follow up the patients and provide dedicated services for testing and treatment, which are not always available elsewhere. On the other hand, coherently with other studies, EDs in addition, even if less well-equipped for STIs, are frequently selected as suitable venues for STIs’ care [21]. This could happen because citizens are unaware of characteristic symptoms or the availability of STI specialized services, or they may inappropriately prefer the ED as a direct access point, especially in situations of perceived urgency, as EDs have no time restrictions and may be closer to the patient’s home [22,23,24].

Furthermore, the insurance status could have played a role in where patients sought care, possibly under the assumption that EDs would treat their condition even if uninsured, however, considering the universal health care system that exists in Italy, treatment is administered freely to everyone.

As only symptomatic patients visit EDs, available clinical studies are frequently focused on a single group or on narrow groups of infections [25,26,27,28], mostly identified through International Classification of Diseases (ICD) codes [19,29,30] and they often pool both adolescents and young adults together [27,31] despite considerable differences.

Given all these premises and the limited availability of information about ED admissions for symptomatic STIs in Italy, this work aims to describe the occurrence of STIs in EDs in the last ten years among the adolescent population in the Piedmont region by using official medical administrative data currently available.

## 2. Materials and Methods

### 2.1. Study Design, Setting and Participants

A retrospective cross-sectional study was carried out in the Piedmont region, the second largest of the 20 Italian regions, covering more than 25,300 km^2^ with a population of over 4.3 million inhabitants, of which nearly 350,000 are aged between 11 to 19.

Information of patients visiting any of the regional EDs between 1 January 2011, and 31 December 2020, were retrieved through the official National ED Information System database.

All admissions to the ED in adolescents between the ages of 11 and 19, likely due to a possible STI and/or major related complications, for instance PID, were initially selected to provide an overview of the issue. Given the low specificity of the information currently available in administrative data sources in the case of complex clinical conditions such as PIDs, to reduce possible identification bias and to ensure a greater precision in the estimations, the analyses focused on symptomatic STIs only (for detailed list see Box 1).

Information on the reason for admission to the ED and the patient’s main circumstances is assigned and checked for consistency upon discharge. Clinical conditions are recorded with an ICD9 code and then each piece of information is anonymized when the data become available for economic and/or epidemiological purposes.

Box 1 Sexually transmitted infections (STIs) codes retrieved. International Classification of Diseases Clinical Modification, ninth revision (ICD9-CM). * Codes selected for the study purposes.

**Sexually Transmitted Infection: ICD9-CM Codes**

Amoebiasis: 006.9

Chlamydia: 077.98; 078.88; 079.98; 099.41; 099.50–099.54; 099.56; 483.1; V73.98 *

Hepatitis A: 070.0; 070.1; 573.3; 771.2

Hepatitis B: 070.2; 070.3; 070.42; 573.3; 771.2; V12.09

Hepatitis C: 070.41; 070.44; 070.51; 070.54; 070.7; 573.3; 771.2; V12.09

Gardnerella vaginalis: 041.89

Giardia: 007.1

Gonorrhea: 098.0; 098.11–098.17; 098.19; 098.37; 098.41; 098.43; 098.50; 098.52; 098.6; 098.7; 098.85; 098.86; 098.89; V01.6 *

Genital Herpes *: 054.10–054.12 *

Genital warts: 078.11; NEC 078.10 *

HIV: 042; 079.53; V08; V72.6; V65.44 *

HPV: 079.4; 795.05; 795.15; 796.74; 796.75 *

Venereal ulcer: 099.2 *

Lymphogranuloma venereum: 099.1 *

Molluscum Contagiosum: 078.0

Mycoplasma hominis: NEC 041.81 *

Phthirus pubis: 132.2

Scabies: 133.0

Shigella: 004.9

Syphilis: 090–099; V65.45 *

Trichomoniasis: 131.0; 131.9 *

Pelvic inflammatory diseases: 614.0–616.0

### 2.2. Ethics

Study data were obtained by accessing official administrative medical records through the universal anonymous patient identity number. The identity number is a ministerial, certified, univocal, non-reversible, anonymous code centrally assigned before data storage. The code allows data management by accredited institutions without any further authorization. As all administrative ministerial data are then made available to authorized bodies in a fully anonymized and de-identified manner, thus ethics committee approval is not required.

### 2.3. Statistical Analyses

To maintain a balance between genders and age groups, three age groups were created, 11 to 13 years, 14 to 16 years, and 17 to 19 years, and then analyzed separately for males and females.

Descriptive analyses were reported for each year between 2011 to 2020 by gender and age groups. Age-specific crude rates, and 95% confidence intervals (CIs), were calculated based on the resident population of the Piedmont region in each year of observation and expressed as number of cases per million population. To account for differences in age distribution within the genders, a direct standardisation with 95% CIs was also calculated to report the overall rate of infection (11 to 19 years of age) by using a 2019 population of the Piedmont region ad data reference.

To account for potential fluctuations in ED hospitalisations over the studied period, the number of ED admissions due to STIs in each year of observation was also considered. An access ratio was then calculated and reported per million ED visits with 95% CI per gender and for each age group.

Differences in the ED access between genders by year of observation and trends over the study period were tested for significance using Chi-square and Cochran–Armitage tests. The significance was set at α < 0.05.

Analyses were performed using the SAS version 9.4 (SAS Institute Inc., Cary, NC, USA).

## 3. Results

An average number of 342,014 adolescents aged 11–19 years old were registered each year as residents in the Piedmont region, between January 2011 and December 2020 without major variations over time (males N = 176,446; females N = 165,568).

The overall number of ED accesses registered during the same period was 1,219,075, and 339 of these were specifically due to STIs, which accounted for a ratio of 27.8 requests every 100,000 accesses.

**Figure 1:**
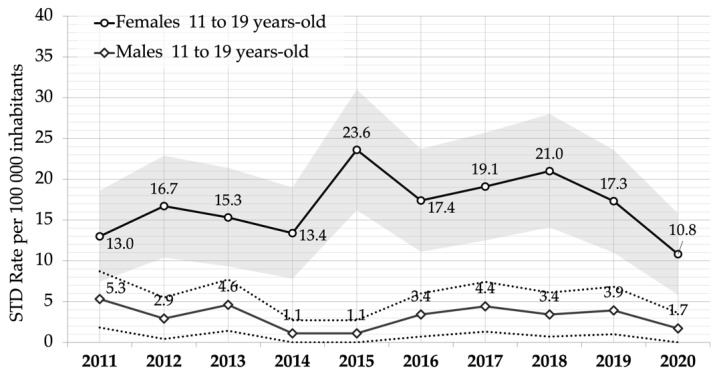
Sexually transmitted infections (STIs) rate per 100,000 inhabitants and 95% confidence intervals (grey and dotted areas), in 11 to 19-year-old adolescents by gender. Year 2011–2020, Piedmont region, Italy.

**Figure 2:**
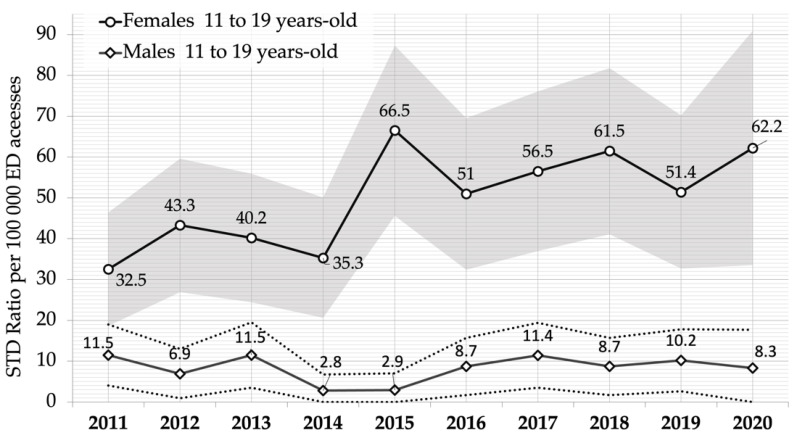
Ratio of sexually transmitted infections (STIs) admissions to the ED per 100,000 visits and 95% confidence intervals (grey and dotted areas), in adolescents aged 11–19 years, by sex. Year 2011–2020, Piedmont region, Italy.

Figure 1 and Figure 2 show the standardized overall ten-year trend for STIs’ rates in 11 to 19-year-olds, and for 11 to 19-year-olds’ ED access ratio by gender.

Infections were significantly far more frequent in females than in males throughout the whole period, and a drop observed in 2014 (13.4; 95% CI 7.8–19.0 in girls and 1.1; 95% CI 0.2–2.7 in boys) was followed by a nearly twofold increase in 2015 in girls (23.6; 95% CI 16.2–31.0).

The age adjusted trend increases until 2018, but in girls only (21.0; 95% CI 14.1–28.0 per 100,000 residents; *p* < 0.001), after which it declined to 10.8 (95% CI 5.8–15.8) in 2020 (Figure 1).

As with the population rate, the same trend was observed for ED visits due to STIs (Figure 2). Infections were significantly less frequent in boys throughout the whole period, which showed an increase in 2015–2019, with a subsequent reduction in 2020. The trend in girls, on the other hand, increased significantly, almost doubling (from 32.5 admissions every 100,000 visits, 95% CI 18.6–46.4) in 2018 (61.5, 95% CI 41.1–81.8). It also showed a deflection in 2019, with a rise in 2020 to the same values observed in 2018 (62.2 admissions, 95% CI 33.5–90.9), showing a general upward trend.

Table 1 reports the number of retrieved infections and the age-specific population rate of ED visits for STIs every 100,000 inhabitants, with 95% CI, per year and by gender.

The lowest rate was observed in males of 11 to 13 years of age, increasing in the 14 to 16 years old group, reaching up to the highest values in the oldest group.

Rates were always significantly higher in girls when compared to their male peers in each year of observation and along the whole period.

In the 17 to 19 years old group, the STI infection rate for females was more than twice to five times higher throughout the whole period. Girls showed a slight increase over the whole period from 25.99 (95% CI 15.5–43.6) in 2011 to 42.98 (95% CI 28.9–64.0) cases per 100,000 in 2019. The highest age-specific STIs’ access rate of 58.3 (95% CI 41.3–82.3) cases per 100,000 inhabitants was observed in the female group of 17 to 19 years of age in 2015. The year 2020 showed a drop in cases of STIs.

As for the girls, the boys also showed the highest rates in the 17 to 19 years old group. In males, the number of infections remained relatively low with fluctuation between 2015 and 2020 resulting in a slight non-significant trend from 15.9 (95% CI 8.3–30.1) in 2011 and 5.0 (95% CI 1.7–14.8) in 2019.

Table 2 reports the number of all ED visits that occurred and the age-specific visits ratio for STIs per year and gender. Overall yearly visits to EDs were significantly higher in males in the 11 to 13 and in 14 to 16 years old group than in girls of the same age, while it was almost equal in the oldest group.

While looking at the overall number of ED visits, a slight decline in both genders can be observed from 2011 (n = 61,662 and n = 73,619, in girls and boys) to 2019 (n = 56,375 and n = 68,638, respectively), followed by a collapse in 2020 (n = 28,263 and n = 36,509).

Similarly, for the observed population rate, the highest age-specific STIs’ visits ratio was observed in the female group from 17 to 19 years of age in 2015 at 151.5 (95% CI 107.3–213.8) admissions per 100,000 visits. The ratio remained quite high in females, at around 120 admissions per 100,000 with a drop in both overall ED visits and STIs’ cases in the year 2020, registering a ratio of 113.54 (95% CI 65.0–198.4) per 100,000 visits.

When looking at the trend of STIs by diagnosis (Table 3), the most frequent causes of ED access were Genital Herpes with a variation of 24.8 admissions every 100,000 ED visits in 2013 to 63.7 in 2020, and Genital Warts in girls. While in males the most frequent cause of ED access was Gonorrhea with a ratio of 1.6 to 8.6, respectively, for the same years. The variation that occurred between 2013 and 2015 in the overall trend in the female sex can also be noticed for the occurrence of the two most prevalent conditions, Genital Herpes and Genital Warts.

## 4. Discussion

The aim of this study was to describe the 10-year trend of sexually transmitted infections among teenagers aged between 11 and 19 years. For this purpose, administrative databases on ED accesses were used.

An increase in the girls’ trend of ED admissions for STIs among 11 to 19-year-olds in Piedmont over the last decade has been reported.

This study showed that, although boys visit EDs more frequently overall, visits for STI-related conditions are more frequent in girls. According to the WHO, younger women and adolescent females are more prone to acquiring STIs because of different reasons, mainly behavioral and not only because of a biological susceptibility [2,17].

While women regularly visit their gynecologist, who can provide immediate help when needed, men, who do not receive the same attention, are likely to leave many of these problems undiagnosed. Previous studies highlighted how female students are more likely to be involved with older partners during their first experience of sexual intercourse than males are, while male students are more likely to have had their first experience of sexual intercourse with a younger partner [32], thus representing a possible cause of this STI frequency difference.

Additionally, in recent years, behavior and opinions about sexuality have changed. Both casual and homosexual sex and acceptance of sexual freedom have increased for boys and girls, thus exposing them to more risks [33].

Our study further showed that infection rates tend to increase steadily with increasing age, which is in line with previous similar research. Infections occurring among 11 to 13-year-olds confirm the early sexual debut of this population, and STIs mostly affect older adolescents, in particular those aged 17 to 19, where sexual activity is also supposed to be likely to increase [33].

Recent national studies report that some Italian adolescents have their first sexual experiences by the age of 13, as this is the period when many individuals will start exploring their sexuality and developing relationships with others [34]. According to the SELFY (Sexual and Emotional Life of Youths) Study, the median age of the first sexual, non-complete experience is 15.5 for boys and 16.5 for girls [33]. It is also well known that early age at sexual debut increases the risk of STI infections.

Adolescents are at higher risk due to their relative physical, emotional, and cognitive immaturity and show a tendency toward more frequent risky behaviors [35]. Moreover, they sometimes approach adulthood with mixed, negative and confusing messages about sexuality, often intensified by the embarrassment and the silence of parents and teachers on this issue [36].

This finding raises questions about the effectiveness of actual health promotion and sexual education interventions offered in schools.

School-based programs represent a significant opportunity to reach many adolescents before they become sexually active. They may offer a structured, protective, and supportive environment to develop knowledge, attitudes, skills, and values. These will empower them to realize their health and well-being, and to establish respectful and positive relationships [36].

In Italy, because of the absence of national planning in the distribution of sexual education, interventions in schools are unevenly distributed among Italian regions. Documents related to the planning, implementation, and evaluation of these activities are lacking as well.

Moreover, this type of education remains very much tied to a health-based tradition of risk and disease prevention, with sporadic and non-continuous interventions offered. A few times, a holistic view was applied using the new framework of Comprehensive Sexuality Education (CSE), contributing to the balanced development of the person and allowing diversity to be valued and supported [37].

As for the type of infections diagnosed in EDs, according to other available international studies, data from the Piedmont population showed that Genital Herpes was reported to be the most frequent infection in girls and it also seems to be on the rise [38,39]. In fact, the CDC’s 2021 guidelines reported an increase in the number of cases of Herpes, both HSV-1 and HSV-2. The CDC also recommended a prevention approach based mainly on person-to-person counselling to foster condom use to reduce the risk of male-to-female transmission, particularly because infections are often asymptomatic in males [20].

In contrast and coherently to what was reported in other countries, Genital Warts, caused by the papillomavirus, showed a slight decrease over time [30]. This is a reduction that could most likely be a direct and visible effect of the mass HPV vaccination strategy adopted in our country along with many other countries in Europe [40,41,42,43,44,45], which deserves further investigation.

The Italian Ministry of Health began to provide the HPV vaccinations in the year 2007, with them being first offered free of charge to 11-year-old girls and subsequently extended to other birth cohorts and to 11-year-old males in 2018. Our data represent a concrete indication of the positive results of this preventive choice. In addition, if the expected vaccination coverage is maintained, the observed HPV infections trend will continue to decrease, thus also influencing the occurrence of cervical cancer. An effect that will be assessable in the near future [46].

As for the male population, in accordance with international surveillance, the most common infection has been reported to be Gonorrhea [47].

Gonorrhea, as well as Syphilis and Venereal Lymphogranuloma, are widespread infections among men, and are reported to be more frequent among men who have sex with men (MSM) [48]. These results would further explain the difference observed in our study highlighting the urgency to rapidly investigate this issue in the older population, and to assess whether specific prevention measures are needed to address this emerging problem in the school-age population.

Similarly, the near-absence of other infections–documented as a priority in terms of incidence (Chlamydia, Syphilis, and Trichomoniasis) by some epidemiological sources–highlights the need to improve the effectiveness of our detection systems, as many of these infections are asymptomatic, and thus risk delaying the request for intervention [49].

In addition, preliminary data (not reported in this paper) showed an alarming volume (N = 2670 only in 2020) of ED visits due to non-specific pelvic organ problems (salpingitis, ovaritis, vaginitis, vulvovaginitis, ulceration of the vulva). Studies report many cases of patients who presented to the ED with complaints of the genitourinary tract and lower abdomen, in whom targeted screening revealed a sexually transmitted infection [50,51]. Unfortunately, the data source used in this study does not allow us to deepen these aspects.

It would be helpful to understand whether, as reported by an increasing number of studies, we are facing a phenomenon of under-use of screening programs or an unexpressed care need, which therefore deserves further comprehension.

Finally, it would be necessary to investigate why adolescents would rather choose EDs, despite the fact that more specific and dedicated services to provide suitable care and treatments have been developed and are offered freely in Italy.

### Study Limitations

This study has some limitations that should be taken into consideration. The first issue is related to the source of information used. The administrative data are complex and sometimes unclear, as these sources originate with a different purpose than an epidemiological one. Such information may be under-represented and may produce incomplete, inaccurate, and potentially tainted data due to coding problems or systematic compilation errors, which have already been reported elsewhere [52,53,54].

Additionally, it is difficult to compare the results with other studies, even with ample literature regarding sexually transmitted infections accessible, as only a few other studies were structured through a similar approach.

To better trust administrative data reports, rates obtained from administrative sources could be juxtaposed with national information notified by clinical and microbiology centers, to allow comparison and to assess any deviations, primarily in mandatory reporting of infections.

## 5. Conclusions

According to other international studies, our results support the need to place more attention on the screening and early diagnosis of STIs in adolescents, particularly in the female population.

Furthermore, it should be noted that infections detected in EDs represent only a proportion of the overall STIs, as a large proportion of them remain undiagnosed.

Longitudinal data allow us to better investigate population needs and to identify specific needs in subgroups, despite the administrative information being less accurate than the clinical information.

To provide a more solid picture, it is recommended to go beyond regional borders by analyzing the issue at the national level, thus increasing cross-country comparisons. Sizing the phenomenon with epidemiological data leads to realizing how the different groups of adolescents approach the issue and eventually the stigma around STIs.

Qualitative investigations on the reasons for preferring EDs over other more suitable services could allow the targeting of more tailored health education activities based on health literacy, empowerment, and self-care strategies.

Deepening adolescents’ awareness about the available STI diagnosis and treatment services, improving their risk perception, and their familiarity with sexuality will be useful to increase knowledge on the barriers to access and to the use of specialized dedicated services.

Finally, the effect of the pandemic on infections deserves further investigation. As COVID-19 has introduced uncertainty and difficulty in interpreting the 2020 data, it would be useful to explain whether the relative STI increase was due to a decrease in overall ED use, or whether lockdown constraints affected the observed infection trends.

## Figures and Tables

**Table 1:** Emergency department (ED) visits, absolute frequency, age-specific rate per 100,000 inhabitants for sexually transmitted infection (STIs), in 11 to 13, 14 to 16 and 17 to 19-year-old adolescents, by gender, years 2011 to 2020, Piedmont region, Italy.

Gender	Year	11 to 13-Year-Olds		14 to 16-Year-Olds		17 to 19-Year-Olds	
		STI Visits	Rate (95% CI)	STI Visits	Rate (95% CI)	STI Visits	Rate (95% CI)
Females	2011	1	1.83 (0.3–10.4)	6	11.29 (5.2–24.6)	14	25.99 (15.5–43.6)
	2012	-	-	9	16.70 (8.8–31.7)	18	33.62 (21.3–53.1)
	2013	3	5.37 (1.8–15.8)	2	3.64 (1.0–13.3)	20	36.99 (23.9–57.1)
	2014	1	1.80 (0.3–10.2)	4	7.22 (2.8–18.6)	17	31.27 (19.5–50.1)
	2015	1	1.79 (0.3–10.2)	6	10.82 (5.0–23.6)	32	58.29 (41.3–82.3)
	2016	1	1.80 (0.3–10.2)	9	16.12 (8.5–30.6)	25	45.25 (30.7–66.8)
	2017	3	5.37 (1.8–15.8)	7	12.61 (6.1–26.0)	22	39.44 (26.1–59.7)
	2018	2	3.57 (1.0–13.0)	7	12.67 (6.1–26.2)	26	46.88 (32.0–68.7)
	2019	1	1.78 (0.3–10.1)	4	7.23 (2.8–18.6)	24	42.98 (28.9–64.0)
	2020	1	1.77 (0.3–10.0)	5	9.01 (3.9–21.1)	12	21.64 (12.4–37.8)
Males	2011	-	-	-	-	9	15.82 (8.3–30.1)
	2012	-	-	-	-	5	8.83 (3.8–20.7)
	2013	2	3.40 (0.9–12.4)	3	5.14 (1.8–15.1)	3	5.20 (1.8–15.3)
	2014	-	-	1	1.68 (0.3–9.5)	1	1.72 (0.3–9.8)
	2015	-	-	-	-	2	3.39 (0.9–12.4)
	2016	1	1.68 (0.3–9.5)	2	3.39 (0.9–12.4)	3	5.01 (1.7–14.7)
	2017	-	-	3	5.12 (1.7–15.0)	5	8.10 (3.5–19.0)
	2018	2	3.37 (0.9–12.3)	3	5.11 (1.7–15.0)	1	1.66 (0.3–9.4)
	2019	2	3.34 (0.9–12.2)	2	3.38 (0.9–12.3)	3	5.04 (1.7–14.8)
	2020	1	1.67 (0.3–9.5)	2	3.38 (0.9–12.3)	-	-

Age-specific rate per 100,000 inhabitants; STI, sexually transmitted infections.

**Table 2:** Absolute year frequency of emergency department (ED) visits and age-specific sexually transmitted infection (STIs) ratio, per 100,000 ED visits, in 11 to 13, 14 to 16 and 17 to 19-year-old adolescents, by gender, years 2011 to 2020, Piedmont region, Italy.

Gender	Year	11 to 13-Year-Olds		14 to 16-Year-Olds		17 to 19-Year-Olds	
		ED Visits	Ratio (95% CI)	ED Visits	Ratio (95% CI)	ED Visits	Ratio (95% CI)
Females	2011	18,326	5.46 (1.0–30.9)	18,915	31.72 (14.5–69.2)	24,421	57.33 (34.2–96.2)
	2012	18,360	-	18,790	47.90 (25.2–91.0)	23,099	77.93 (49.3–123.2)
	2013	18,644	16.09 (5.5–47.3)	18,945	10.56 (2.9–38.5)	22,814	87.67 (56.8–135.4)
	2014	19,018	5.26 (0.9–29.8)	19,551	20.46 (8.0–52.6)	22,595	75.24 (47.0–120.5)
	2015	17,963	5.57 (1.0–31.5)	18,971	31.63 (14.5–69.0)	21,126	151.47 (107.3–213.8)
	2016	17,486	5.72 (1.0–32.4)	18,040	49.89 (26.3–94.8)	20,557	121.61 (82.4–179.5)
	2017	17,600	17.05 (5.8–50.1)	18,388	38.07 (18.4–78.6)	20,355	108.08 (71.4–163.6)
	2018	18,087	11.06 (3.0–40.3)	17,850	39.22 (19.0–80.9)	20,614	126.13 (86.1–184.8)
	2019	18,184	5.50 (1.0–31.2)	17,854	22.40 (8.7–57.6)	20,337	118.01 (79.3–175.5)
	2020	8637	11.58 (2.0–65.6)	9057	55.21 (23.6–129.2)	10,569	113.54 (65.0–198.4)
Males	2011	26,447	-	22,434	-	24,738	36.38 (19.1–69.1)
	2012	26,031	-	21,637	-	22,741	21.99 (9.4–51.5)
	2013	25,908	7.72 (2.1–28.2)	21,690	13.83 (4.7–40.7)	21,775	13.78 (4.7–40.5)
	2014	26,519	-	22,275	4.49 (0.8–25.4)	21,903	4.57 (0.8–25.9)
	2015	25,134	-	21,314	-	21,433	9.33 (2.6–34.0)
	2016	25,332	3.95 (0.7–22.4)	21,388	9.35 (2.6–34.1)	21,737	13.80 (4.7–40.6)
	2017	25,907	-	21,487	13.96 (4.8–41.1)	22,118	22.61 (9.7–52.9)
	2018	25,858	7.73 (2.1–28.2)	21,042	14.26 (4.9–41.9)	21,927	4.56 (0.8–25.8)
	2019	25,787	7.76 (2.1–28.3)	21,201	9.43 (2.6–34.4)	21,650	13.86 (4.7–40.7)
	2020	12,869	7.77 (1.4–44.0)	11,488	17.41 (4.8–63.5)	12,152	-

Age-specific rate per 100,000 inhabitants; STI, sexually transmitted infections.

**Table 3:** Emergency department (ED) visits, absolute frequency and cause specific ratio per 100,000 ED visits, by gender and year of observation. Period 2011–2020, Piedmont region, Italy.

	STI/Year	2011	2012	2013	2014	2015	2016	2017	2018	2019	2020
		N (Ratio)	N (Ratio)	N (Ratio)	N (Ratio)	N (Ratio)	N (Ratio)	N (Ratio)	N (Ratio)	N (Ratio)	N (Ratio)
Females	Chlamydia	-	-	-	-	-	-	-	-	-	-
	Genital Herpes	17 (27.57)	20 (33.2)	15 (24.83)	20 (32.7)	28 (48.23)	28 (49.93)	28 (49.7)	30 (53.05)	28 (49.67)	18 (63.69)
	Genital Warts	3 (4.87)	6 (9.96)	8 (13.24)	1 (1.63)	8 (13.78)	4 (7.13)	1 (1.77)	4 (7.07)	1 (1.77)	-
	Gonorrhea	1 (1.62)	1 (1.66)	1 (1.66)	1 (1.63)	1 (1.72)	1 (1.78)	2 (3.55)	1 (1.77)	-	-
	Inguinal granuloma	-	-	1 (1.66)	-	1 (1.72)	2 (3.57)	-	-	-	-
	HIV	-	-	-	-	1 (1.72)	-	1 (1.77)	-	-	-
	Ulcer venereal	-	-	-	-	-	-	-	-	-	-
Males	Chlamydia	-	1 (1.61)	-	-	-	-	-	-	-	-
	Genital Herpes	2 (3.14)	1 (1.61)	1 (1.60)	1 (1.58)	1 (1.66)	1 (1.72)	2 (3.43)	1 (1.71)	1 (1.71)	-
	Genital Warts	2 (3.14)	2 (3.21)	1 (1.60)	1 (1.58)	-	-	-	1 (1.71)	1 (1.71)	1 (3.3)
	Gonorrhea	2 (3.14)	1 (1.61)	5 (8.60)	-	1 (1.66)	5 (8.61)	5 (8.57)	4 (6.83)	4 (6.85)	2 (6.6)
	Inguinal granuloma	2 (3.14)	-	-	-	-	-	1 (1.71)	-	1 (1.71)	-
	HIV	-	-	-	-	-	-	-	-	-	-
	Ulcer venereal	1 (1.57)	-	1 (1.60)	-	-	-	-	-	-	-

N, number of cause-specific STIs observed in Eds; ratio, STIs proportion every 100,000 ED visits.

## Data Availability

Datasets used in the current study are not publicly available due to embedded legal policies. Data are managed and were provided by the Epidemiology Unit, Regional Health Service, Local Health Unit TO3, Piedmont region, Italy. Any access to datasets is granted to allowable bodies upon written agreement between involved institutions. Requests can be addressed to the attention of the director of the service.
